# 

**Published:** 1898-04

**Authors:** 


					﻿Southern Medical Record
A Monthly Journal of Medicine and Surgery.
Vol. XXVIII.	ATLANTA, GA., APRIL, 1898.	No. 4.
BERNARD WOLFF, M. D., Editor.
Associate Editors :
A. W. GRIGGS, M. D.
j. McFadden gaston. m.d.
WILLIS F. WESTMORELAND, M.D.
B. M. ZETTLER, Business Manager.
Entered at the Post-office at Atlanta, Ga., as Second-class Matter.
The Foote & Dj, Co., Atlanta, Ga.
				

